# Supplementary material for: Ultrastaging of the Parametrium in Cervical Cancer: A Clinicopathological Study
Source: Cancers (Basel). 2023 Feb 9;15(4):1099. doi: 10.3390/cancers15041099 (PMC9954180; doi:10.3390/cancers15041099)
Supplement: Supplementary file 1 [file cancers-15-01099-s001.zip › cancers-2139564-supplementary.pdf]

Ultrastaging of parametrium in cervical cancer: a clinicopathological study

Supplementary Table S1. Performance of parametrial ultrastaging in predicting pelvic lymph node metastasis.

**Statistic**	**Value**	**95% CI**
Sensitivity	16.67%	0.42% to 64.12%
Specificity	100.00%	86.28% to 100.00%
Positive Likelihood Ratio		
Negative Likelihood Ratio	0.83	0.58 to 1.19
Disease prevalence	19.35%	7.45% to 37.47%
Positive Predictive Value	100.00%	
Negative Predictive Value	83.33%	77.76% to 87.73%
Accuracy	83.87%	66.27% to 94.55%
